# Prevalence and Genetic Diversity of Parasites in Humans and Pet Dogs in Rural Areas of Los Ríos Region, Southern Chile

**DOI:** 10.3390/pathogens14020186

**Published:** 2025-02-13

**Authors:** Daniel Sanhueza Teneo, Tamara Venegas, Francisca Videla, Cedric B. Chesnais, Carlos Loncoman, Guillermo Valenzuela-Nieto

**Affiliations:** 1 Instituto de Inmunología y Parasitología, Facultad de Medicina, Universidad Austral de Chile, Valdivia 5110566, Chile; tamara.venegas01@alumnos.uach.cl (T.V.); francisca.videla@alumnos.uach.cl (F.V.); 2 TransVIHMI, Institut de Recherche pour le Développement (IRD), INSERM Unité 1175, Montpelier University, 34090 Montpellier, France; cedric.chesnais@ird.fr; 3 VirionLab, Instituto de Bioquímica y Microbiología, Facultad de Ciencias, Universidad Austral de Chile, Valdivia 5110566, Chile; carlos.loncoman@uach.cl; 4 Facultad de Ciencias para el Cuidado de la Salud, Universidad San Sebastián, Valdivia 509000, Chile; guillermo.valenzuela@uss.cl

**Keywords:** parasites, zoonosis, public health, southern Chile, prevalence

## Abstract

Parasitic infections pose a significant global health burden, affecting millions of people worldwide. Despite their importance, studies integrating human and animal parasitology to understand transmission pathways are scarce. This study, conducted between August 2022 and April 2023, aimed to investigate the prevalence of parasites in humans and domestic dogs in the Los Rios Region, southern Chile, and explore the risk factors associated with parasitism. A total of 291 human participants provided fecal and blood samples for parasitological and serological analyses, while 92 fecal samples from owned dogs were analyzed. The detection of intestinal parasites employed microscopy and molecular techniques, including next-generation sequencing (NGS). Anti-*Toxocara canis* and anti-*Echinococcus granulosus* antibodies in humans were assessed using ELISA. Socioeconomic surveys explored the risk factors associated with parasitism. The results showed a parasite prevalence of 39% in humans and 40% in dogs. Anti-*Toxocara canis* IgG antibodies were detected in 28.2% of humans. Zoonotic subtypes of *Giardia duodenalis* and *Blastocystis* sp. were identified. Thus, the results of our study indicate a high prevalence of intestinal and extraintestinal parasites in the human population of our region. Furthermore, our findings underscore the significant risk of zoonotic transmission of parasites from companion animals. This study contributes to the understanding of parasite transmission dynamics in southern Chile and has implications for public health policy and practice. The results emphasize the importance of considering the connection between human, animal, and environmental health to develop effective control strategies and mitigate the impact of parasitic infections.

## 1. Introduction

Parasitic infections represent a significant global burden of disease, particularly in developing countries, but they are also of concern in developed nations due to increasing human migration [1]. Worldwide, around 1.5 billion people are infected with geohelminths—soil-transmitted helminths—including, as main species, *Ascaris lumbricoides*, *Trichuris trichiura*, and hookworms (*Ancylostoma duodenale* and *Necator americanus*) [2]. In addition, nearly one-third of the world human population is infected with *Toxoplasma gondii* [3]. Intestinal protozoa transmitted through the fecal–oral route are also highly prevalent, with *Blastocystis* sp., *Entamoeba* spp., *Giardia duodenalis* (syn. *Giardia lamblia*), and *Cryptosporidium* spp. being the most common [4,5]. 

In Chile, research on the prevalence of parasitic infections in the human population remains scarce. However, localized studies indicate substantial rates of infection. For instance, in the city of Colina, *Blastocystis* sp. showed the highest prevalence (41.3%) among children, followed by *Entamoeba coli* (17%) and *G. duodenalis* (12.6%) [6]. A similar high prevalence was described in a study conducted in Lluta Valley in Arica, in the north of Chile, where *G. duodenalis* showed a prevalence of 30%, followed by *Enterobius vermicularis* (28%), *Entamoeba histolytica* (15.3%), and *Endolimax nana* (27.3%) [7]. However, in recent years, national-level public health interventions, such as expanded urban drinking-water coverage (from 52% in 1950 to 99.9% in 2014) and improved sewage infrastructure, have significantly reduced cases of enteric infections [8] (Crespo 2018). For example, in Talca, Chile, the prevalence of pathogenic parasitic species in preschoolers and schoolchildren between 1980 and 2008 decreased, with *G. duodenalis* prevalence decreasing from 18.45% to 5.53%, *T. trichiura* from 7.6% to 0.3%, *A. lumbricoides* from 10.03% to 0.11%, *E. histolytica* from 2.3% to 0.81%, and *Taenia* sp. from 1.25% to 0.12%. Conversely, the prevalence of *Blastocystis* sp. dramatically increased from 7–61% to 72.97% during the same period [9].

The geographical distribution of parasites in Chile exhibits a pattern characterized by unevenness. The different environmental and climatic conditions present in the country, such as coastal or rural sectors, humid or dry climates, and multiple types of soil, provide favorable conditions for the development of certain parasitic species that require specific conditions for their survival. This spatial distribution is reflected, for instance, in the high prevalence of reported cases of hydatidosis in regions dedicated to sheep farming such as Aysén and Los Lagos [10] (Instituto de Salud Publica de Chile 2023), as well as in the case of *Dibothriocephalus latus*, which has been diagnosed in people from lakeside communities in the regions of Los Ríos, Los Lagos, and Aysén [11,12], with an observed prevalence in humans ranging from 0.2% to 3.4% until 2004 [13]. 

Despite these findings, recent studies on human parasitic infections in the Los Ríos Region are limited. In 1997, riparian communities of the Valdivia River basin exhibited high prevalence rates of *Blastocystis* sp. (50.5%), *Entamoeba coli* (24.6%), *Endolimax nana* (16.9%), and *G. duodenalis* (23.8%) [14]. Helminths such as *A. lumbricoides* (15.5%), *T. trichiura* (12.7%), and Trichostrongylidae gen. sp. (0.2%) were also detected in 1995 in communities bordering the Valdivia River [15]. Furthermore, serological evidence of zoonotic parasites such as *Toxocara* spp. (25.4%) of seroprevalence in the locality of Niebla highlights their importance in the region [16]. This parasite causes the syndromes of visceral larva migrans (VLM) and ocular larva migrans (OLM) in humans, emphasizing the importance of quantifying the prevalence of zoonotic parasites in our region. 

In Chile, 75.9% of households have pets, mainly dogs and cats, and about 28.7% of them do not deworm their pets every six months [17]. In Los Angeles (Chile), 60% of public squares and parks are contaminated with zoonotic parasites, with *Toxocara* spp. (9.2%) standing out as the most relevant [18]. In Chile, the prevalence of intestinal parasites diagnosed in dogs ranges from 32.8% to 51.6%, according to studies carried out in the canine population of the municipalities of three different regions. Thus, in the region of the capital Santiago, the main parasites found in dogs are the protozoa *Giardia* spp. (23.3%) and *Isospora* spp. (13.3%), followed by the nematode *Toxascaris leonina* (8.3%). Meanwhile, in the south of the country (Biobío and Los Ríos Regions), the main parasites found correspond with the helminths *Ancylostoma caninum*, *Capillaria* sp., *Uncinaria stenocephala*, *Trichuris vulpis*, and *Toxocara canis* [19,20,21]. Another parasite of dogs present in Chile is *Echinococcus granulosus*, the causative agent of hydatidosis in humans, a disease under epidemiological surveillance in the country [10]. A study conducted on dogs from a northern region (Coquimbo) detected an overall prevalence of 7.2% [22].

Among zoonotic diseases, giardiasis, caused by *Giardia duodenalis*, serves as a model for understanding cross-species transmission. Based on molecular studies, it has been shown that there are different host-specific genetic assemblages, with assemblages A and B infecting humans and overlapping with livestock and pet animals [23]. *Blastocystis* sp., another zoonotic parasite, exhibits complex subtype (ST) diversity, with 44 STs identified worldwide to date [24]. ST1-ST8, ST12, ST14, and ST16 have been found to infect/colonize humans and animals, ST9 has been found only in humans, and the rest of the STs have only been identified in other hosts. ST4, ST5, ST6, ST7, and ST8 could have zoonotic transmission to humans; these subtypes have been found to infect rodents, pigs, birds, and non-human primates [25]. ST1-ST4 are the most common in the human population, accounting for 90% of cases, with ST3 being the most common worldwide. The pathogenicity of this parasite shows a differentiation according to the ST that is present in the host, so this infection is not always observed in association with symptoms. ST1, 2, and 4 have been associated with gastrointestinal symptoms, while no association has been found in other studies [26]. 

This study provides updated data on the prevalence of intestinal parasites and, for the first time, conducts molecular analyses to identify and describe the subtypes of *G. duodenalis* and *Blastocystis* sp. in the Los Rios Region of Chile. It also examines the seroprevalence of IgG anti-*Toxocara canis* and IgG anti-*Echinoncoccus granulosus* antibodies in humans and investigates the prevalence of intestinal parasitic infections in domestic dogs. By using an integrated approach, this research aims to enhance our understanding of the geographic dynamics of parasitic infections in southern Chile, with the aim of implementing transmission control measures adapted to the epidemiologic setting of southern Chile.

## 2. Materials and Methods

### 2.1. Study Area

The study area was the Los Ríos Region, Chile, bounded by latitudes 39°15′ S and 40°33′ S, extending from the Pacific Ocean to the border with Argentina, with an area of 18,429 km^2^. This territory is divided into 12 communes. According to the 2017 census, the total population of the region is 384,837 inhabitants, with Valdivia being the largest city and the regional capital, with 127,750 inhabitants. Of this population, 275,786 people live in urban areas, while 109,051 live in rural areas (https://www.bcn.cl/siit/nuestropais/nuestropais/region14 accessed on 1 March 2022) [27]. Each area has its own environment and peculiarities, including variations in soil composition due to different geological origins. To better understand the characteristics of this region, we divided it into the following three subregions:

Subregion 1: The coast is the region closest to the Pacific Ocean and is mainly maritime. There are a significant number of rivers (Valdivia, Chaihuin, Lingue, and Bueno, among others) that flow into the ocean in the form of large estuaries. The cities or towns of this subregion are associated with economic and cultural activities related to rivers and the sea.

Subregion 2: The central zone is part of the so-called intermediate depression and is mainly associated with forestry and agriculture, of which 70.7% corresponds with forest plantations, 19.1% with fodder, and 6.2% with cereals. In terms of livestock, there are 629.385 cattle, 117.830 sheep, and 34.532 pigs in the region, most of which are located in this subregion.

Subregion 3: This is the area closest to the Andes Mountains. In winter, it has the lowest temperatures in the region and, due to its 10 lakes, it has different ecological characteristics, with cases of parasites such as *Dibothriocephalus* sp. or *Trichinella spiralis* not reported in the other subregions.

### 2.2. Research Ethics Committee Approval

The study obtained the necessary permissions for its realization from the Research Ethics Committee of Los Ríos Health Service (Ord. N°084 25.03.2022). Adult participants signed the “Information and informed consent document”. Minors were asked for their consent and that of their parents/guardian by means of the “Information document and informed assent for study in minor users” signed by the participant and the “Parental informed consent document for a study in minor users” signed by the parents/guardian.

### 2.3. Collection and Processing Methodologies

The 12 communes that make up the region were invited to participate in this research through the health department of each commune; 7 of them accepted the invitation. Each commune is divided into localities, each of which has a rural health center (for the purposes of the study, we call them “sampling points”) (Table 1).

### 2.4. Collection Methodology

The study was conducted between August 2022 and April 2023. At each sampling point, a maximum of 20 participants were included. For the diagnosis of intestinal parasites, each participant was provided with a 35 mL container of PAF (phenol, alcohol, and formaldehyde) fixative and a 15 mL conical-bottomed tube with 8 ml of 70% ethanol, along with oral and written instructions for proper sample collection. Each participant collected three stool samples on alternate days (days 1, 3, and 5). On the final day of sampling (day 5), participants were instructed to provide an additional sample in the 70% ethanol-containing falcon tube for molecular techniques. Stool samples in PAF were stored at 2–5 °C, while those in ethanol were stored at −20 °C until further processing. 

For the serological testing of IgG antibodies against *T. canis* and *E. granulosus* in humans, sampling sites were randomly selected, with up to 20 participants per site. Blood samples were collected by venipuncture using tubes without an anticoagulant and centrifuged at 3000 rpm for 5 min. The serum was extracted and then frozen at −20 °C until the ELISA test was performed. Additionally, each participant completed a survey to identify potential socioeconomic factors associated with the prevalence of parasitic infections. 

Participants who consented to include their dogs in the study were provided with a container of 15 mL of PAF fixative and were asked to collect a single sample of 5 grams of dog feces. In addition, a second sample should have been left in the falcon tube with 70% ethanol, which was used for molecular techniques.

### 2.5. Processing of Stool Samples 

The stool samples, both human and canine, were processed using the modified Burrows method (PAFS) [28], and each sample was examined using light microscopy at 10×, 40×, and 100×. 

### 2.6. Sample Processing Using ELISA 

The anti-IgG antibodies *T. canis* and anti-*E. granulosus* were measured in the Parasitology Laboratory of Facultad de Medicina, Universidad Austral de Chile, in the city of Valdivia. The kits used were NOVATEC kits manufactured by Bordier Affinity Products S.A. The tests were performed according to the manufacturer’s instructions. Samples with indeterminate results were sent to the national reference laboratory at the Institute of Public Health, Santiago, Chile, for confirmation. 

### 2.7. Genetic Diversity of Giardia duodenalis and Blastocystis sp.

Samples from human or canine individuals showing the presence of parasites such as *Blastocystis* sp. or *Giardia duodenalis* in EPSD were selected to identify the subtypes of these parasitic organisms. For this purpose, DNA was extracted from the samples previously stored in alcohol at −20 °C using the Qiagen DNeasy PowerSoil Pro Kits extraction kit, which was modified according to the kit protocol with the addition of Proteinase K to facilitate cell rupture and DNA extraction. The polymerase chain reaction (PCR) technique was then applied and, finally, the samples were sent to Austral-omics for dideoxynucleotide sequencing. Raw sequencing data were curated using Geneious software (Version 11.0.18). Forward and reverse sequences were subjected to a BLAST analysis via the Blastocystis typing database (https://pubmlst.org/bigsdb?db=pubmlst_blastocystis_seqdef) (accessed on 1 March 2022). In the case of *Giardia duodenalis*, the sequences used correspond to B-giardin gene were obtained from Dos Reis et al., 2023 [29]. Both parasites’ sequences were mapped to the best match based on the BLAST and downloaded from the database using the Geneious mapper with the highest sensitivity setting, performing fine-tuning iterations up to 100 times. A consensus sequence was generated with a strict threshold of 75%, using only bases with a Phred quality score above 20. All consensus sequences were aligned with sequences available from the NCBI database (alignment attached), using Geneious for the multiple sequence alignment (MSA). The software automatically determined the sequence direction. The alignment type was set to global alignment with free-end gaps, using a 65% similarity cost matrix (5.0/−4.0) with a gap open penalty of 15 and a gap extension penalty of 5. Refinement iterations were set to 50. A phylogenetic tree was constructed using the Jukes–Cantor genetic distance model and the neighbor-joining method, with *Proteromonas lacertae* (Accession: PLU37108) as the outgroup. Bootstrap resampling was performed with 100 replicates using a random seed of 535,190 and a support threshold of 50%. The resulting phylogenetic tree was visualized and edited using Figtree software (v1.4.4). The sequences generated by these analyses were deposited in GenBank and the following access numbers were provided for the *Blastocystis* sp. sequences: PV013372, PV013373, PV013374, PV013375, PV013376, PV013377, PV013378, PV013379, PV013380, PV013381, PV013382, PV013383, PV013384, PV013385, PV013386, PV013387, PV013388, PV013389, PV013390, PV013391, PV013392, PV013393, PV013394, PV013395, PV013396, PV013397, PV013398, and PV013399; and PV023177, PV023178, and PV023179.2.8 for *Giardia duodenalis*.

### 2.8. Statistical Analysis 

A descriptive analysis of the data was performed using the chi-squared method, and a statistical significance level of 0.05 was established. To determine the magnitude of the observed relationship, the odds ratio (OR) was used with a 95% confidence interval. GraphPad Prism 9 and IBM SPSS Statistics (Statistical Package for the Social Sciences) were used for the analysis of the data obtained.

## 3. Results

### 3.1. Results of Serial Stool Parasitological Analysis

**Figure 1:**
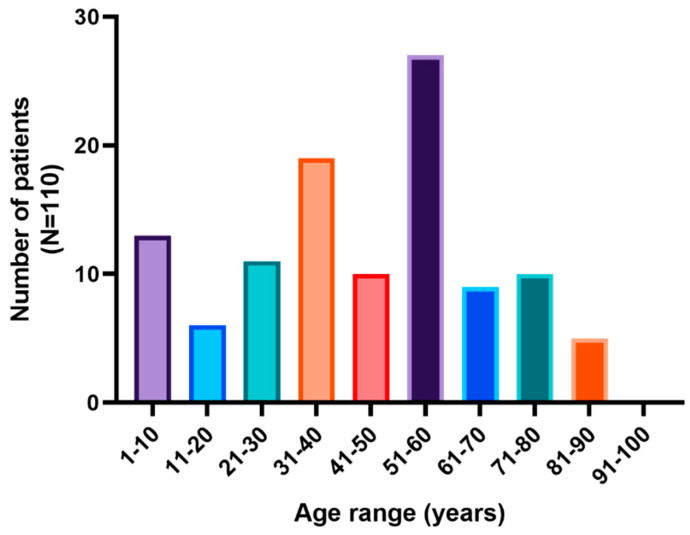
Number of patients with intestinal parasites by age range.

A total of 291 human fecal samples were analyzed. The age of the participants ranged from 1 year to 95 years. The overall prevalence of intestinal parasites in the region was 39%. Among individuals with confirmed parasitic infections, 55.55% were female and 45.45% were male. The age of infected participants ranged from 1 to 87 years, with a mean of 44 years (Figure 1). Regarding the number of parasite taxa per individual, 62.9% of cases involved monoparasitism, while 37.1% exhibited polyparasitism. 

**Figure 2:**
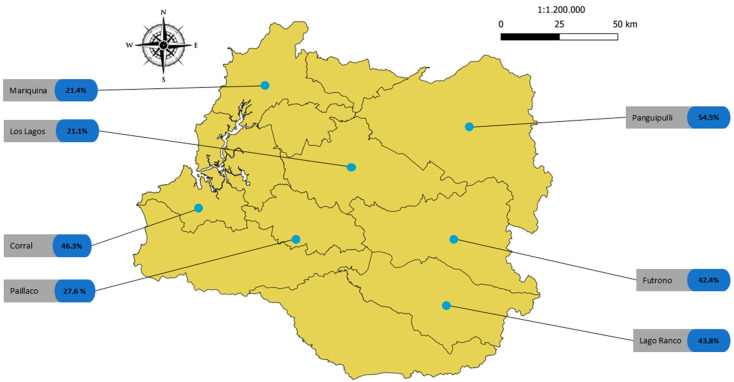
Prevalence of human intestinal parasites in each commune of the Los Rios Region. Source: based on the image of Region de Los Rios by Biblioteca del Congreso Nacional de Chile.

By commune, the highest prevalence of intestinal parasites was recorded in Panguipulli, where 54.5% of the samples tested positive (18 of 33). The lowest prevalence was recorded in the commune of Los Lagos (21.1%). In subregion 1 (coast), the commune of Corral had the highest prevalence of human intestinal parasites (46.3%), with San Juan being the sampling site with the highest prevalence (67%). In subregion 2 (central), Paillaco was the commune with the highest prevalence of intestinal parasites (27.6%). In subregion 3 (mountains), Panguipulli was the commune with the highest prevalence (54.5%), and Coñaripe was the sampling site with the highest prevalence (85.7%) (Figure 2).

**Figure 3:**
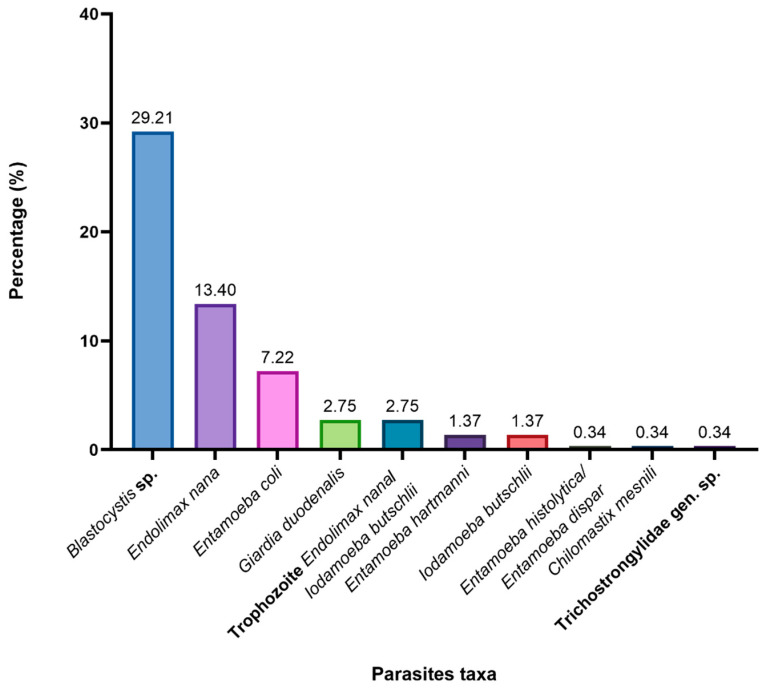
Prevalence of intestinal parasites in humans.

It was observed that intestinal protozoa were the most common agents of parasitic infections in humans. Notably, only one case of nematode infection, caused by Trichostrongylidae gen. sp., was identified in our study. By parasite, the highest prevalence was observed for *Blastocystis* sp. (29.2%), followed by *Endolimax nana* (13.4%), Entamoeba coli (7.2%), and *G. duodenalis* (2.8%) (Figure 3).

#### Statistical Analysis of Serial Stool Parasitological Analysis

There was a statistically significant relationship between the sex of the participant and being infected with parasites (χ^2^ = 5.195; *p*-value of 0.023). Men had a 1.78 times higher risk of having intestinal parasites than women (OR = 1.775; 95% confidence interval (CI) = 1.081–2.912). Also, the results of the survey showed that exclusively consuming safe drinking water had a statistically significant relationship with the presence of parasites (χ^2^ = 4.492; *p*-value = 0.034), being a protective factor against the presence of *Blastocystis* sp. (OR = 0.565; 95% CI = 0.332–0.961).

A comparison between subregions showed that there was a statistically significant difference in the number of positive samples between the coast and the center (χ^2^ = 4.991; *p*-value = 0.025), with twice the risk of having a positive sample in the coast than in the center (OR = 2.213; 95% CI = 1.095–4.470). There was also a statistically significant difference in the number of positive samples between the mountain and the center subregions (χ^2^ = 8.966; *p*-value = 0.003), having more than twice the risk of parasites in the mountain subregion (OR = 2.616; 95% CI = 1.380–4.961). Thus, the subregion with the lowest probability of having a sample with any parasite was subregion 2 (center) compared with the others two subregions. 

### 3.2. Serologic Study Results for IgG Anti-Toxocara canis and Anti-Echinococcus granulosus Antibodies

**Figure 4:**
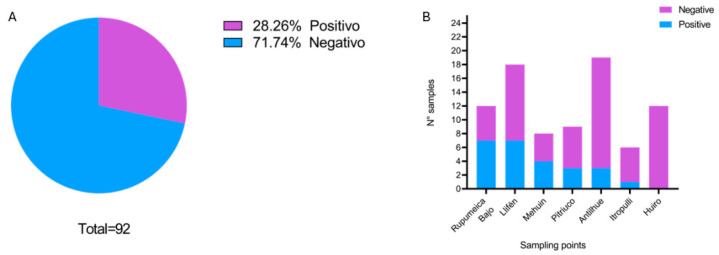
(**A**) Prevalence of anti-*T. canis* antibodies in human serum samples; (**B**) number of samples with anti-*T. canis* antibodies per sampling site.

A total of 92 samples were analyzed by ELISA to detect the presence of both anti-*T. canis* and anti-*E. granulosus* from the following 8 different sampling points: Pitriuco (Lago Ranco), Rupumeica Bajo (Lago Ranco), Llifén (Futrono), Antilhue (Los Lagos), Itropulli (Paillaco), Mehuín (Mariquina), Huiro (Corral), and Isla del Rey (Corral). In the case of IgG anti-*E. granulosus* antibodies, 97.8% (n = 90) of the samples were negative, while 2 samples gave indeterminate results; they were sent for confirmation to the Institute of Public Health and they remain indeterminate. On the other hand, regarding the prevalence of anti-*T. canis* antibodies, 28.2% (n = 26) of the samples were positive, with Rupumeica Bajo (Lago Ranco) being the location with the highest seroprevalence (58.3%) (Figure 4). 

### 3.3. Results of Parasitological Examination of Dogs Feces 

**Figure 5:**
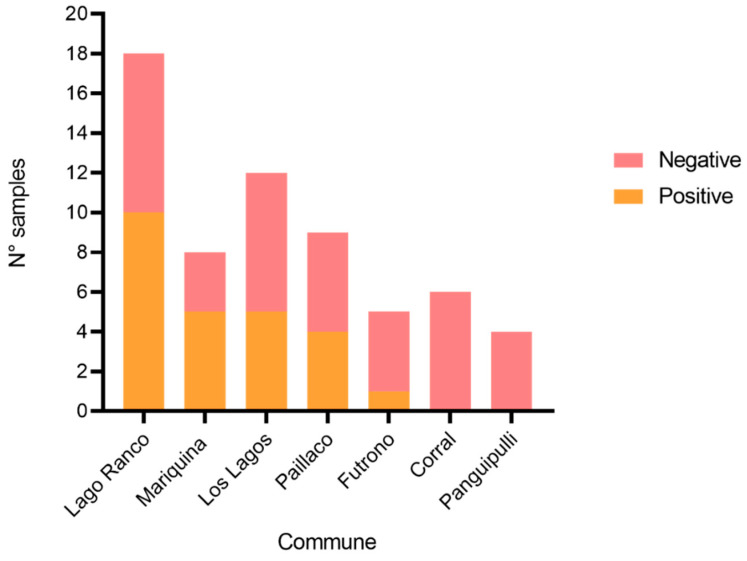
Number of samples with intestinal parasites in dogs by commune.

In total, 62 fecal samples were analyzed. An overall prevalence of 40% (n = 25) was observed. When analyzed by commune, it was observed that Mariquina had the highest prevalence of parasites (62.5%), followed by Lago Ranco (55%), Paillaco (44.4%), Los Lagos (41.6%), and Futrono (20%). On the other hand, in Panguipulli and Corral, no parasitic elements were found (Figure 5). 

**Figure 6:**
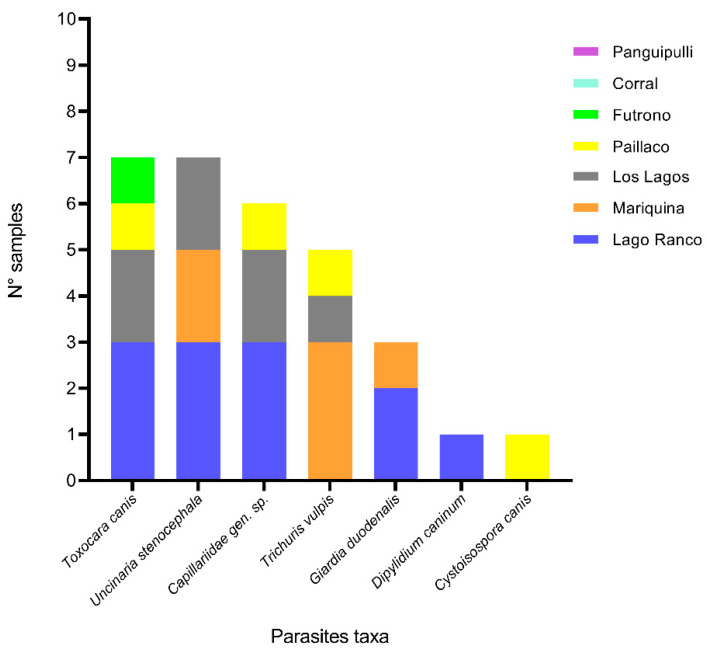
Number of samples with different parasite taxa found in feces of dogs by commune.

By parasites, the prevalence described in our study was *T. canis* (28%), *U. stenocephala* (28%), *Capillaridae* gen. sp. (24%), *T. vulpis* (20%), *Giardia duodenalis* (12%), *Dipylidium caninum* (4%), and *Cystoisospora canis* (4%). Most of these parasites were distributed in several communes of the region (Figure 6).

### 3.4. Phylogenetic Analysis of Blastocystis sp. and Giardia duodenalis

**Figure 7:**
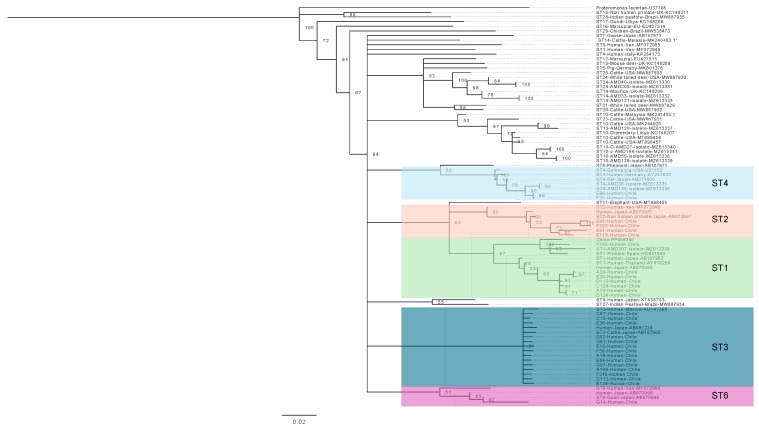
Phylogenetic tree of *Blastocystis* sp., showing the position of our samples in the subtypes 1, 2, 3, 4, and 6. Bootstrap support values appear on the branches.

For the analysis of *Blastocystis* sp. subtypes, 28 human samples were used. These samples were diagnosed as positive for this parasite using light microscopy, as previously described. These samples yielded 28 study sequences (consensus sequences), which were compared with 58 sequences published in the scientific literature. The result of this analysis showed that subtypes 1, 2, 3, and 4 had been described and subtype 6 had been described for the very first time in Chile (Figure 7). 

**Figure 8:**
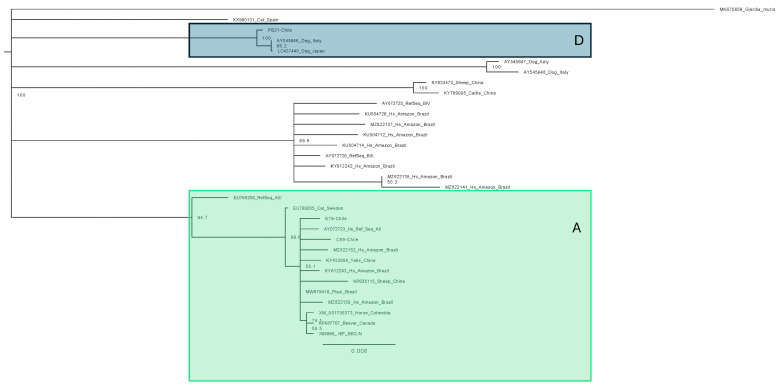
Phylogenetic tree of *G. duodenalis*, showing the position of our samples in the assemblage A and D. Bootstrap support values appear on the branches.

In the case of *G. duodenalis*, it was possible to work with 3 samples (2 human and 1 canine) previously identified as positive for this parasite using light microscopy because good-quality amplification was not achieved for the other samples presenting this parasite. These 3 consensus sequences were compared with 29 sequences from literature. The results of our human samples showed that they belonged to assembly A and the dog sample belonged to assembly D (Figure 8).

**Figure 9:**
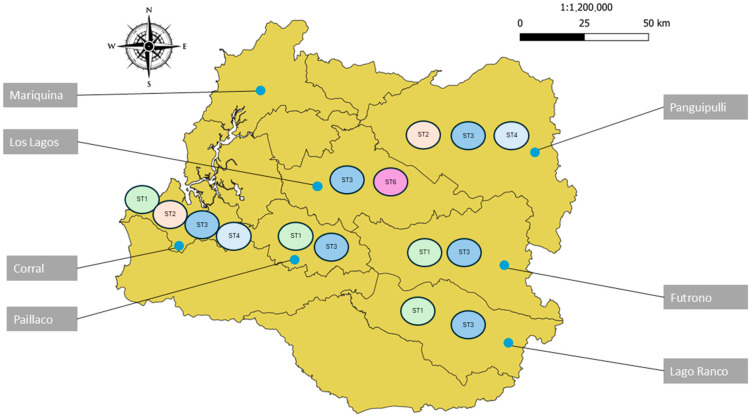
Geographic distribution of *Blastocystis* sp. subtypes in the different communes of the Los Rios Region. Source: Based on the image of Region de Los Rios by Biblioteca del Congreso Nacional de Chile.

The subtypes described for both parasites were distributed in the coastal, central, and mountain areas without any geographical predilection (Figure 9).

## 4. Discussion 

The overall prevalence of intestinal parasites in humans in our study was 39%, closely aligning with a similar study conducted in Santiago, Chile, which reported a prevalence of 37.76% [30]. However, this prevalence was lower than that reported in other studies conducted in Latin America. For instance, two studies in Venezuela reported prevalences of 53.02% and 67.36%, respectively [31,32]. In Bolivia, the prevalence reached 73.60% [33].

**Figure 10:**
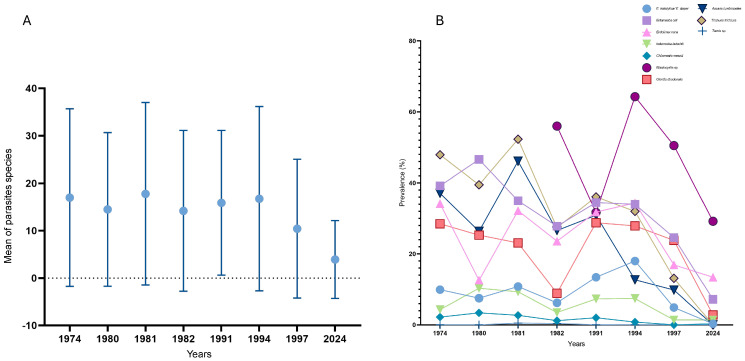
(**A**): Overall mean and SD of parasites over time. (**B**): Temporal variation in prevalence of intestinal parasites.

In the Los Ríos Region of Chile, the prevalence of parasites drastically decreased over time, as illustrated in Figure 10A. Furthermore, a shift in the most prevalent parasites in the region was observed. In earlier studies from 1974, 1981, and 1982, helminths such as *T. trichiura* and *A. lumbricoides* were the most prevalent, with a rate reaching up to 50%. In contrast, our study identified no helminths apart from a single sample containing Trichostrongylidae gen. sp., representing a prevalence of just 0.34% (Figure 10B) [14,34,35,36,37,38,39]. However, it should be emphasized that the studies of 1974 [39], 1981 [36], 1991 [38], 1994 [37], and 1997 [14] were carried out using preschool and school populations of our region. This population-specific focus could introduce a bias that complicated direct comparisons with our results, which included a broader demographic. This difference might also explain the lower prevalence observed in the 1982 study [35], which was conducted using an adult population (Figure 10A,B). 

Our study found a decrease in the prevalence of all protozoan species, with their current prevalences at historical lows. Given of the transmission mode of these protozoa, primarily via contaminated water or food, the most plausible explanation for this trend is an improvement in drinking-water quality [40]. One study developed an Environmental Performance Index (EPI) for the category of drinking water for 180 different countries. The results concluded that Chile is the leader in Latin America in terms of sanitation and drinking-water quality [41]. In line with this, our statistical analyses revealed that the exclusive consumption of safe drinking water was a protective factor against the presence of *Blastocystis* sp., the most prevalent parasite in the region. This finding underscores the critical role of improved water quality in reducing parasitic infections in the region. 

In the case of anti-*E. granulosus* antibodies, no positive samples were detected in our study. This result could reflect the decreasing prevalence of this parasite in the region. Between 2011 and 2021, the Los Rios Region reported the second lowest case numbers in southern Chile, with only five confirmed cases [10].Consequently, it is challenging to observe a high prevalence of antibodies for this parasite without prior clinical suspicion. 

In contrast, the prevalence of anti-*T. canis* antibodies was 28.26%, consistent with a study conducted in a rural area near Valdivia in 2016, which reported a prevalence of 25.4% [16]. This similarity could be attributed to the widespread presence of stray dogs across the region. We observed no significant differences in prevalence among the coastal, central, and mountainous subregions. It is important to point out the high prevalence of this zoonotic parasite in our region, which shows that humans with positive serology had consumed eggs of *T. canis* originating from its definitive host, the domestic dog (*Canis familiaris*). This highlights the public health importance of addressing zoonotic parasitic infections in Los Ríos.

For owned dogs in the region, the overall prevalence of intestinal parasites was 40%. This was slightly lower than the 51.6% described in Santiago, Chile [42], but higher than the 32.8% observed on an island near the city of Valdivia, Chile [21]. Among the detected parasites, *T. canis* (28%), *G. duodenalis* (12%), and *Dipylidium caninum* (4%) were prominent, all of which are of significant public health concern due to their potential to cause serious diseases in humans such as intestinal disorders, malabsorption syndrome, or syndromes like visceral larva migrans (VLM) and ocular larva migrans (OLM) [43,44,45]. Supporting this idea of zoonotic transmission of parasites in our region, we observed a high prevalence of *U. stenocephala*, Capillaridae gen. sp., and *T. vulpis*, also described as parasites with zoonotic potential [42]. 

Regarding the genetic diversity of *Blastocystis* sp., we found subtypes 1, 2, 3, and 4, which are the ones that circulate the most in America and have been previously described in our country. However, we describe ST 6 for the first time in Chile, which had already been described in Latin America in Argentina, Brazil, and Colombia [46]. This subtype has been described as having a very low prevalence in Latin America, being present in 0.39% of all human samples analyzed and in 2.59% of animal samples, confirming the possible spillover of this strain from animals to humans. It has been statistically correlated with *Phasianus colchicus* (pheasant) (*p*-value = 0.00266) [47], so this strain could be associated with rural farming, which would be consistent with the rural area of sampling in this study.

The results of the study indicated a high prevalence of intestinal parasites and anti-*Toxocara canis* antibodies in humans. Furthermore, the prevalence of intestinal parasites in pet dogs was also high. Most of the subtypes described for *Blastocystis* sp. and *Giardia duodenalis* are associated with zoonotic transmission. This aspect is crucial for comprehending parasitic transmission in the Los Rios Region, Chile.

In conclusion, our findings provide an updated overview of parasitic infections in the region, highlighting the importance of conducting integrative studies to understand parasite transmission dynamics. By elucidating the routes of transmission, we can better comprehend the epidemiological reality of parasitic infections in our region. Such knowledge is crucial for implementing targeted control measures to mitigate the risks of transmission between humans and from animals to humans. 

## Figures and Tables

**Table 1:** All sampling points for each commune in the Los Rios Region. Subregion Coast: Mariquina and Corral; Center: Paillaco and Los Lagos; Mountains: Lago Ranco, Futrono, and Pangupulli.

Sampling Points in Los Rios Region, Chile								
Commune	Mariquina	Corral	Paillaco	Los Lagos	Lago Ranco	Futrono	Pangupulli	
ID of Sampling Points	Mehuin	Isla del Rey	Pichirropulli	El Salto	Pitriuco	Maihue	Panguipulli	Cayumapu
	Missisipi	Chaihuin	Reumén	Pellinada	Riñinahue	Curriñe	Coñaripe	Huitag
	Chan chan	Huape	Santa Filomena	Malihue	Illahuapi	Chabranco	Choshuenco	Lago Neltume
	Iñipulli	San Juan	Aguas Negras	Antilhue	Calcurrupe	Arquilhue	Neltume	Puerto Fuy
		Las Coloradas	Santa Rosa	Riñihue	Pocura	Llifén	Liquiñe	
		Huiro	Itropulli	Las Huellas	Rupumeica bajo	Loncopán	Melefquen	
			El Llolly			Huapi	Bocatoma	
			La Luma				Pocura	

## Data Availability

The data presented in this study are available on request from the corresponding author due to privacy and legal restrictions related to human participants.
